# IL-22 and IDO1 Affect Immunity and Tolerance to Murine and Human Vaginal Candidiasis

**DOI:** 10.1371/journal.ppat.1003486

**Published:** 2013-07-11

**Authors:** Antonella De Luca, Agostinho Carvalho, Cristina Cunha, Rossana G. Iannitti, Lucia Pitzurra, Gloria Giovannini, Antonella Mencacci, Lorenzo Bartolommei, Silvia Moretti, Cristina Massi-Benedetti, Dietmar Fuchs, Flavia De Bernardis, Paolo Puccetti, Luigina Romani

**Affiliations:** 1 Department of Experimental Medicine and Biochemical Sciences, University of Perugia, Perugia, Italy; 2 Istituto Superiore di Sanità, Roma, Italy, Italy; 3 Microbiology, S. Maria della Misericordia Medical Center, Perugia, Italy; 4 Innsbruck Medical University, Innsbruck, Austria; University of Massachusetts Medical School, United States of America

## Abstract

The ability to tolerate *Candida albicans*, a human commensal of the gastrointestinal tract and vagina, implicates that host defense mechanisms of resistance and tolerance cooperate to limit fungal burden and inflammation at the different body sites. We evaluated resistance and tolerance to the fungus in experimental and human vulvovaginal candidiasis (VVC) as well as in recurrent VVC (RVVC). Resistance and tolerance mechanisms were both activated in murine VVC, involving IL-22 and IL-10-producing regulatory T cells, respectively, with a major contribution by the enzyme indoleamine 2,3-dioxygenase 1 (IDO1). IDO1 was responsible for the production of tolerogenic kynurenines, such that replacement therapy with kynurenines restored immunoprotection to VVC. In humans, two functional genetic variants in *IL22* and *IDO1* genes were found to be associated with heightened resistance to RVVC, and they correlated with increased local expression of IL-22, IDO1 and kynurenines. Thus, IL-22 and IDO1 are crucial in balancing resistance with tolerance to *Candida*, their deficiencies are risk factors for RVVC, and targeting tolerance via therapeutic kynurenines may benefit patients with RVVC.

## Author Summary

This study disentangles resistance and tolerance components of murine and human *C. albicans* vaginal infection and introduces the challenging notion of a disease due to a defective tolerance mechanism. Vulvovaginal candidiasis (VVC) and recurrent VVC (RVVC) are two forms of disease that affect a large number of otherwise healthy women. Uncomplicated VVC is associated with several predisposing factors, whereas RVVC, marked by idiopathic recurrent episodes, may be virtually untreatable. Despite a growing list of recognized risk factors, further understanding of anti-*Candida* host defense mechanisms in the vagina is needed to optimize vaccine development and immune interventions to integrate with, or even replace, antifungal therapy. Indeed, medical treatments that increase host resistance, such as antifungals, are highly effective for individual symptomatic attacks but do not prevent recurrences and there is concern that repeated treatments might induce drug resistance. As tolerance mechanisms are not expected to have the same selective pressure on pathogens, new drugs that target tolerance will provide therapies to which low-virulence pathogens, such as *C. albicans*, will not develop resistance. This study provides a proof-of-concept that targeting tolerance via therapeutic kynurenines may benefit patients with RVVC.

## Introduction

*Candida* species are the causative agents of vulvovaginal candidiasis (VVC) and recurrent VVC (RVVC), two forms of disease that affect a large number of otherwise healthy women [1], [2]. Uncomplicated VVC is associated with several predisposing factors, including antibiotic and oral contraceptive usage, hormone replacement therapy, pregnancy and uncontrolled diabetes mellitus, and it usually responds to treatment. In contrast, RVVC, marked by idiopathic recurrent episodes, may be virtually untreatable. Despite a growing list of recognized risk factors, further understanding of anti-*Candida* host defense mechanisms in the vagina is needed to optimize vaccine development [3], [4] and immune interventions to integrate with, or even replace, antifungal therapy.

Colonization of the vaginal mucosa by the fungus induces both humoral and Th immunity [5]–[7], with the contribution of epithelial [8] and dendritic cells [6]. Acquired Th1 [9]–[11] and Th17 [12] immunity have been described in murine and human VVC. Thus, multiple effector mechanisms of resistance to the fungus are apparently present in VVC. As IL-22 is known to contribute to antifungal resistance at mucosal surfaces by assuring epithelial integrity [13]–[15], and low levels of IL-22 are associated with chronic and recurrent mucosal candidiasis [16]–[19], a role for this cytokine in vaginal immune resistance, beyond the polymorphonuclear neutrophil's (PMNs) response and alarmins production [20], is likely.

In addition to resistance mechanisms that reduce pathogen burden during infection, tolerance mechanisms that protect the host from immune- or pathogen-induced damage have recently emerged in the area of animal immunity [21], [22]. It has been argued that a high rate of infection, but low virulence, should select for host tolerance, whereas the opposite condition should favor resistance [23]. Therefore, it is not surprising that tolerance is a complementary host defense trait that increases fitness in response to low-virulence *C. albicans* in the host-*Candida* symbiosis [24]. Considerable evidence for an association of recurrent episodes of symptomatic infection with immune hyper-reactivity to the fungus [25]–[27] point to the contribution of a deregulated immune reactivity to the pathogenesis of VVC and support a role for immunoregulation in this disease. As a matter of fact, protection from VVC is associated with limited or absent inflammatory responses that will not necessarily cause the elimination of the fungus, whereas symptomatic infection is associated with a heavy vaginal cellular infiltrate of PMNs and a variable degree of fungal presence [8], [28]. A plethora of tolerance mechanisms, despite less clarified than resistance mechanisms, have been described [29], [30]. In murine VVC, CD4^+^ CD25^+^ regulatory T (Treg) cells [31], γ/δ T cells [32] and immunoregulatory cytokines, such as IL-10 and transforming growth factor β, have all been demonstrated. Thus, resistance and tolerance are complementary host antifungal defense mechanisms that likely operate in the vaginal mucosa, where the ability to tolerate the fungus implies immune strategies that favor the induction of non-sterilizing protective immunity in an environment permissive for fungal persistence.

Indoleamine 2,3-dioxygenase 1 (IDO1), the rate-limiting enzyme in tryptophan degradation along the kynurenine pathway [33], is a master regulator of antifungal tolerance at mucosal surfaces [34]. Pathogenic inflammatory responses due to IDO1 deficiency account for the inherent susceptibility of mice to aspergillosis [35] and mucosal candidiasis [36], owing to the unopposed inflammatory responses that compromise the host's ability to efficiently oppose fungal infectivity. By regulating the balance between Th17 and Treg cells, IDO1 may not only contribute to local immune homeostasis but also limit the pro-survival and virulence-promoting activity of IL-17A on fungal cells [37]. A role for IDO1 in the genitourinary system seems likely, because of the intense IDO1-specific staining in a number of tissues from the genitourinary system [38]. There is also evidence for IDO1 involvement in persistent genitourinary *Chlamydia trachomatis* infection [39]. However, whether IDO1 contributes to protective tolerance to *C. albicans* in the vagina is presently unknown.

In the current study, we evaluated the role of IL-22 and IDO1 in murine and human VVC. We used mice with selective deficiency of IL-22 or IDO1 to explore innate and acquired Th/Treg mechanisms of antifungal protection and patients with VVC and RVVC in which common genetic variants in the *IL22* and *IDO1* genes were analyzed and correlated with local cytokine production. We found that genetic deficiencies of IL-22 or IDO1 were associated with VVC in mice, due to impaired resistance and tolerance mechanisms to the fungus. Two functional genetic variants in human *IL22* and *IDO1* were associated with a decreased risk for RVVC and correlated with increased local expression of IL-22, IDO1 and kynurenines. This study demonstrates that IL-22 and IDO1 mediate antifungal resistance and tolerance to *C. albicans* in the vagina and that their deficiencies are risk factors for RVVC.

## Results

### IL-22 mediates resistance and IDO1 restrains inflammation in murine VVC

**Figure 1:**
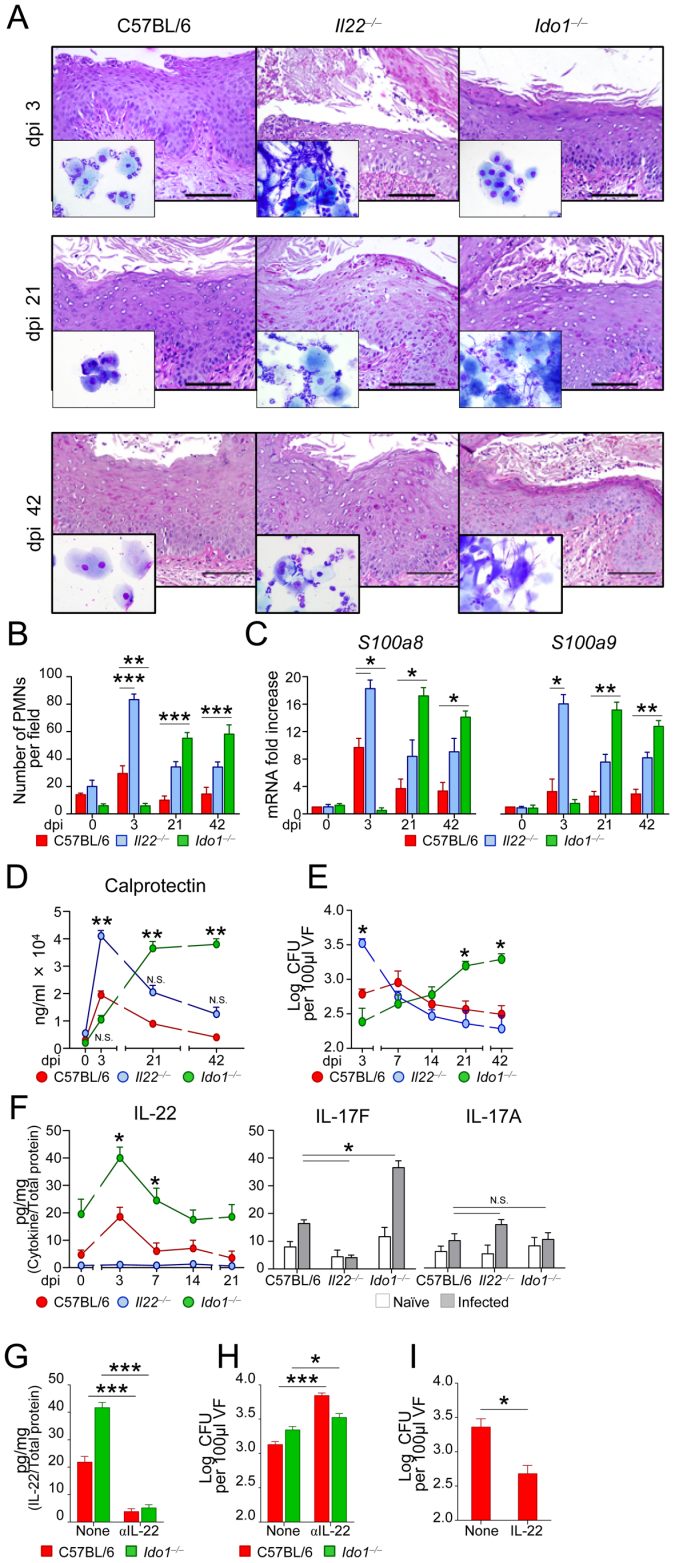
Vaginal candidiasis in IL-22- or IDO1-deficient mice. C57BL/6, IL-22- or IDO1-deficient mice (*n* = 6) were intravaginally inoculated with 5×10^6^ *C. albicans* blastoconidia. (**A**) Periodic acid-Schiff-staining of vaginal sections and inflammatory cell recruitment in vaginal fluids (May–Grünwald Giemsa staining in the insets) at different days post-infection (dpi). Representative images of histology sections and vaginal fluids were acquired with a 40× and 100× objective respectively. Scale bars, 100 µm. (**B**) Polymorphonuclear cells (PMNs) quantification in the vaginal fluids. PMNs were identified by nuclear morphology and enumerated per field at ×100 magnification. Each point represents an individual mouse, and horizontal bar indicates the means. **P<0.01 and ***P<0.001, IDO1- or IL-22-deficient *vs.* wild-type mice at the indicated days. (**C**) *S100a8* and *S100a9* mRNA expression in vaginal tissue by real-time RT-PCR. The mRNA-normalized data were expressed as relative mRNA in IDO1- or IL-22-deficient *vs.* wild-type mice. *P<0.05 and **P<0.01, IDO1- or IL-22-deficient *vs.* wild-type mice at the indicated days. (**D**) Levels of calprotectin during vaginal candidiasis. **P<0.01, IDO1- or IL-22-deficient *vs.* wild-type mice. (**E**) Vaginal fungal burden in mice at different dpi. CFU were quantified by culturing serial dilutions of vaginal fluids (VF) from each mouse and expressed as Log_10_ CFU/100 µl VF ± s.e.m. *P<0.05, IDO1- or IL-22-deficient *vs.* wild-type mice at the days indicated. Data are pooled or representative (histology) from four independent experiments. (**F**) Cytokine levels (pg/mg, cytokine/total proteins for each sample) in the vaginal fluids (at 3 dpi for IL-17F and IL-17A). Results represent mean cytokine levels (± s.e.m.) from samples pooled from three experiments (*n* = 4–6 total samples per group). *P<0.05, IDO1- or IL-22-deficient *vs.* wild-type mice. (**G**) Levels of IL-22 (pg/mg, cytokine/total proteins) and (**H**) fungal growth (at 3 dpi) in mice treated with 300 µg of mAb neutralizing IL-22 or isotype control mAb (None) given intraperitoneally the day of and 1 day after the primary infection. (**I**) Fungal growth (at 3 dpi) in mice treated intravaginally with rIL-22 or PBS (None) the day of and 1 and 2 days after the infection. Pooled data from two experiments (*n* = 6). *P<0.05 and ***P<0.001, treated *vs.* untreated mice. N.S., not significant.

IL-22 and IDO1 are key mediators of resistance and tolerance to *Candida* [13], [36] and other fungi [14], [35] at mucosal surfaces. To assess the role of IL-22 and IDO1 in murine VVC, we intravaginally infected C57BL/6, IL-22- or IDO1-deficient mice with *Candida* blastospores and evaluated resistance to infection in terms of vaginal histopathology, PMN recruitment in vaginal lavages, expression of chemotactic S100A8 and S100A9 proteins, known to mediate PMN migration in murine VVC [8] and local fungal growth. In C57BL/6 mice, histological analysis revealed the presence of fungal and inflammatory cells infiltrating the vaginal parenchyma with signs of epithelial damage at the early stages (Figure 1A). Robust PMN recruitment (Figure 1B and insets in Figure 1A, dpi 3), significant *S100a8* and *S100a9* gene expression (Figure 1C) and calprotectin production (Figure 1D) were also observed. Mice eventually controlled the infection, as indicated by a reduction in fungal burden (Figure 1E), PMN number, *S100a8 and S100a9* expression, calprotectin level and amelioration of inflammatory pathology at 21 and 42 dpi (Figure 1A–D). The course of the infection was different in IL-22- *vs.* IDO1-deficient mice and in those mice *vs.* their respective wild-type counterparts. IL-22-deficient mice were susceptible to *C. albicans* in the early but not late stages of infection, as indicated by signs of vaginal epithelial damage and inflammation, robust PMN recruitment, high *S100a8* and *S100a9* gene expression, calprotectin levels and fungal growth at 3 dpi (Figure 1A–E). An opposite pattern of resistance was observed in IDO1-deficient mice, in which resistance to infection was increased early but not late in infection. Indeed, at days 21 and 42 after the infection, mice were unable to restrict fungal growth and inflammation (Figure 1A–E). Wild-type and IL-22-deficient, but not IDO1-deficient mice, showed resistance to re-infection (Figure S1A in Text S1), a memory response requiring an intact T cell compartment (Figure S1B–E in Text S1). These findings suggest that IL-22 mediates early antifungal resistance, whereas IDO1 is required to restrain inflammation during ongoing infection and to provide antifungal memory.

**Figure 2:**
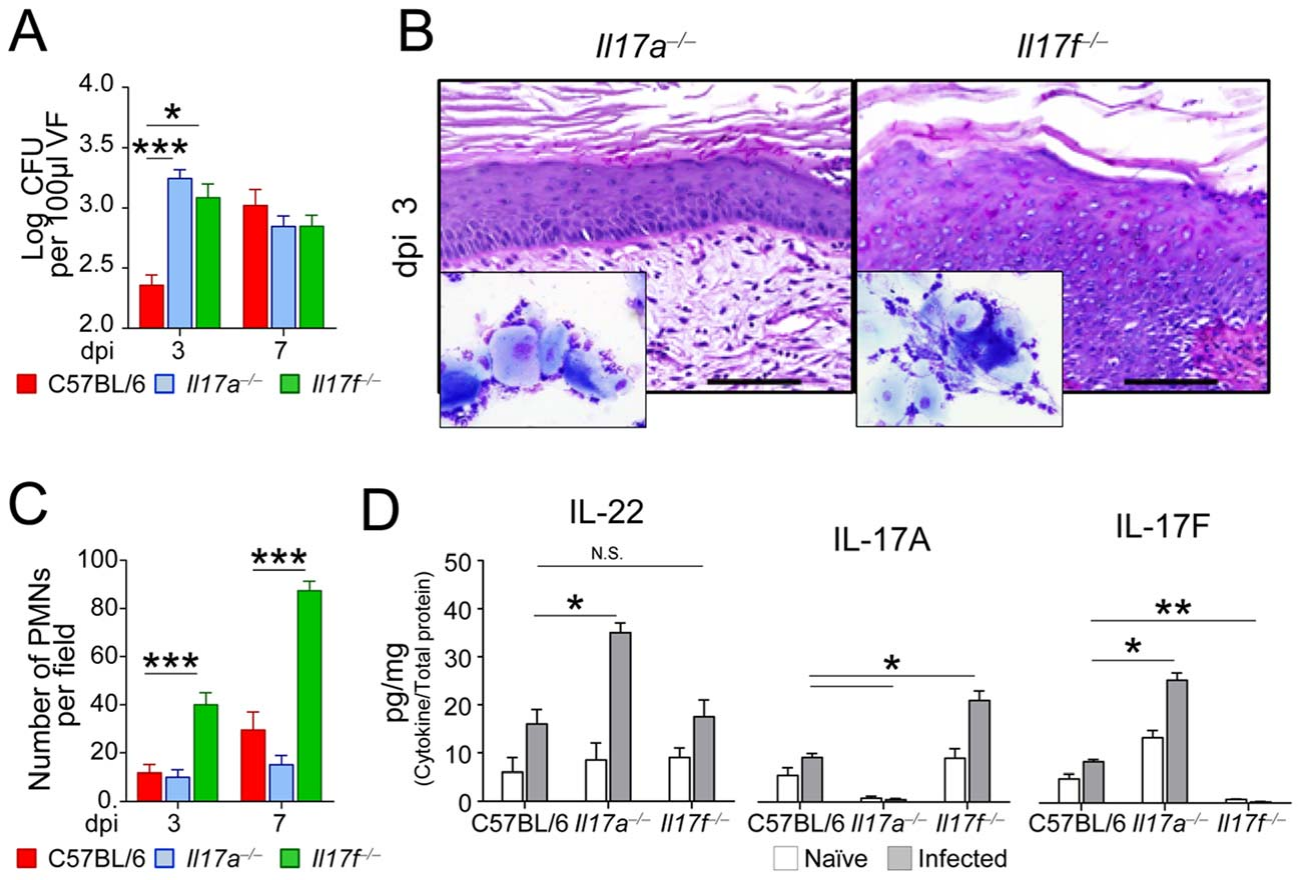
Vaginal candidiasis in IL-17A- or IL-17F-deficient mice. (**A**) Vaginal fungal burden in C57BL/6, IL-17A- or IL-17F-deficient mice (*n* = 6) intravaginally inoculated with 5×10^6^ *C. albicans* blastospores. CFU were quantified by culturing serial dilutions of vaginal fluid (VF) from each mouse at different days post-infection (dpi) and expressed as Log_10_ CFU/100 µl VF ± s.e.m. Pooled data from 3 experiments. (**B**) Histological analysis of periodic acid-Schiff-stained vaginal sections and inflammatory cell recruitment in vaginal fluids (May–Grünwald Giemsa staining in the insets) at 3 dpi. Representative images (out of 3 experiments) of histology sections and vaginal fluids were acquired with a 40× and 100× objective respectively. Scale bars, 100 µm. (**C**) Polymorphonuclear cells (PMNs) quantification in the vaginal fluids at different dpi. PMNs were identified by nuclear morphology and enumerated per field at ×100 magnification. Each point represents an individual mouse, and horizontal bar indicates the means. (**D**) Cytokine levels (pg/mg, cytokine/total proteins for each sample) in the vaginal fluids of naïve or infected (3 dpi) mice. Data are pooled or representative (histology) from 3 independent experiments. * *P*<0.05, **P<0.01, ***P<0.001, knockout *vs.* wild-type mice. N.S., not significant.

To directly prove the role of IL-22, we evaluated levels of IL-22, as well as of companion cytokines, such as IL-17A and IL-17F, in infection and the consequences of IL-22 inhibition or administration. We found elevated levels of IL-22 through the infection in IDO1-deficient mice in which high levels of IL-17F, but not IL-17A, were also higher as compared to wild-type mice (Figure 1F). Blocking IL-22 (Figure 1G) greatly increased fungal growth in IDO1-deficient mice (Figure 1H), whereas exogenous IL-22 decreased fungal growth in wild- type mice (Figure 1I), a finding confirming that IL-22 mediates antifungal resistance in VVC particularly under conditions of IDO1 deficiency. Experiments in IL-17A- or IL-17F-deficient mice confirmed the superior activity of IL-22 *vs.* IL-17A in early anticandidal resistance in VVC. Despite both types of mice show a higher fungal burden early in infection, as compared to wild-type mice (Figure 2A), IL-17F-deficient, more than IL-17A-deficient, mice showed signs of inflammation (Figure 2B) and PMN recruitment (Figure 2C and inset in Figure 2B) associated with high levels of IL-17A (Figure 2D). Thus, confirming recent findings [20], the neutrophil response in vaginal candidiasis occurs independently of IL-22 and IL-17F. Interestingly, the fact that IL-17A binds fungal cells in the vagina and affects fungal growth and morphology [37], may account for the numerous hyphae observed in vaginal fluids from IL-17F-deficient mice (inset in Figure 2B).

### IL-22 is produced by vaginal innate lymphoid cells via AhR

**Figure 3:**
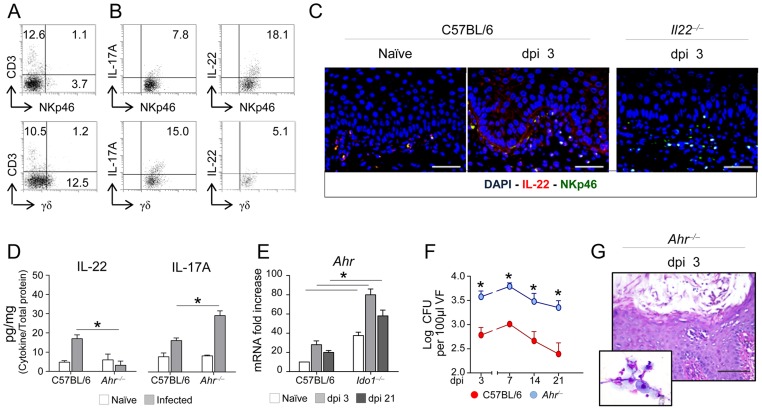
IL-22 is produced by innate lymphoid cells via AhR. (**A**) Numbers of CD3^+^ NKp46^+^ and CD3^+^ γδ T cells in total vaginal cells from naïve C57BL/6. Numbers refer to % positive cells (one representative experiment out of 2). (**B**) IL-22-and IL-17A-producing NKp46^+^ and γδ T vaginal cells. Cells were purified from the vagina of naïve C57BL/6 mice and intracellularly stained for IL-17A or IL-22 before FACS analysis. Numbers refer to % positive cells. (**C**) Vaginal immunohistochemistry of naïve (dpi 0) C57BL/6 or IL-22-deficient mice at 3 days post-infection (dpi). Staining was done with anti- NKp46-PE and anti-IL-22-FITC antibody. Cell nuclei were stained with DAPI (blue). Representative pictures (out of 2 experiments) were taken with a 20× objective. Scale bars, 50 µm. (**D**) IL-22 and IL-17A levels on vaginal fluids (pg/mg, cytokine/total proteins for each sample at 3 dpi) of mice (*n* = 6) intravaginally inoculated with 5×10^6^ *C. albicans* blastoconidia. *P<0.05 AhR-deficient *vs.* C57BL/6 mice. (**E**) *AhR* expression (RT-PCR) in the vagina of infected mice at different dpi. *P<0.05 IDO1-deficient *vs.* C57BL/6 mice. (**F**) Vaginal fungal burden and (**G**) vaginal histology of AhR-deficient mice (*n* = 6) intravaginally inoculated with 5×10^6^ *C. albicans* blastospores. CFU (Log_10_ CFU/100 µl vaginal fluids ± s.e.m.) and vaginal histology (periodic acid-Schiff-staining at 3 dpi) were done at 3 dpi. In the inset, inflammatory cell recruitment in vaginal fluids by May–Grünwald Giemsa staining at 3 dpi. Representative images were acquired with a 40× and 100× objective respectively. Scale bars, 100 µm. Data are pooled or representative (histology) from 2 independent experiments. *P<0.05 AhR-deficient *vs.* C57BL/6 mice.

In gastrointestinal candidiasis, IL-22 is mainly produced by NKp46^+^ NK1.1^low^ cells, an innate lymphoid cell (ILC) subset expressing the aryl hydrocarbon receptor (AhR) [13]. We searched for IL-22-producing NKp46^+^ cells in the vagina of C56BL/6 mice along with γδ T cells, also known to produce IL-22 at mucosal surfaces [40]. Both types of cells, with the predominance of γδ T cells, were present in the vagina, as revealed by FACS analysis (Figure 3A). However, NKp46^+^cells mainly produced IL-22, as shown by intracellular cytokine staining in vitro (Figure 3B) and vaginal immunostaining in vivo (Figure 3C). Indeed, IL-22-producing NKp46^+^ cells expanded in infection in wild-type but not IL-22-deficient mice (Figure 3C). In contrast, γδ T cells produced IL-17A more than IL-22 (about 15% IL-17A^+^ *vs.* 5% IL-22^+^ cells) (Figure 3B). IL-22, but not IL-17A, production was significantly decreased in AhR-deficient mice (Figure 3D), a finding suggesting that IL-22 is produced by vaginal NKp46^+^ via AhR. As a matter of fact, not only was *AhR* expression increased during VVC, particularly in IDO1-deficient mice (Figure 3E) but AhR-deficient mice were also more susceptible to VVC (Figure 3F and G). Thus, much like in the gastrointestinal tract [41], IL-22 produced via AhR may serve as a first-line resistance mechanism against yeast infection at the vaginal level.

### IDO1 is required for development of tolerance in murine VVC

**Figure 4:**
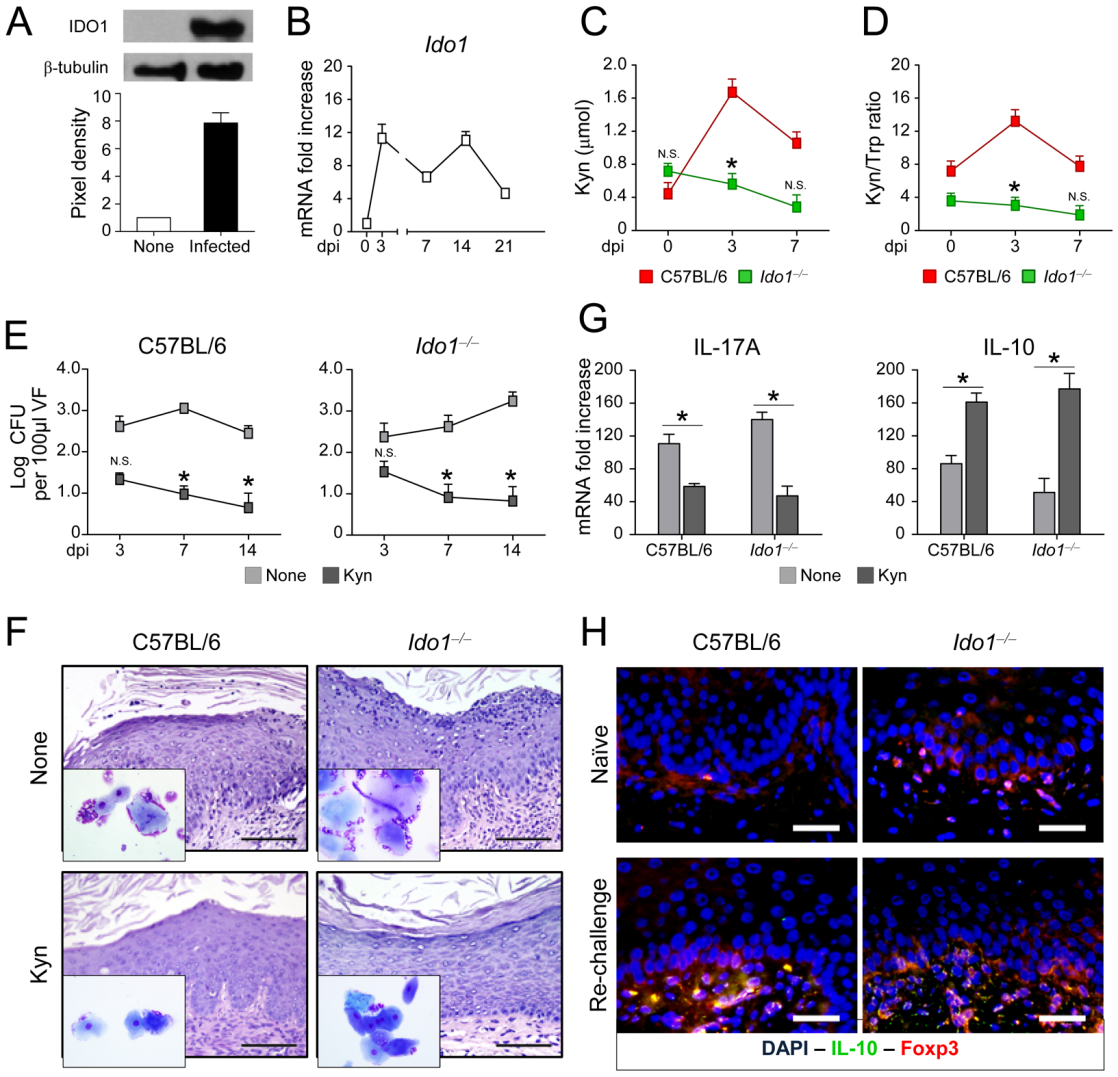
IDO1 and kynurenines mediate tolerance in murine VVC. (**A**) IDO1 protein and (**B**) gene expression in the vagina of C57BL/6 mice (*n* = 4) intravaginally infected with *C. albicans*. Proteins in vaginal cell lysates (3 dpi) were visualized by western blotting with rabbit polyclonal IDO1 specific antibody. Scanning densitometry was done on a Scion Image apparatus. Western blots out of 2 independent experiments and corresponding pixel density ratio normalized against β-tubulin. *Ido1* mRNA expression [normalized to mRNA of naïve (dpi 0) mice] in vaginal tissue (RT-PCR) at different dpi. (**C**) Relative concentrations of kynurenines (Kyn) and (**D**) kynurenine-to-tryptophan (Kyn/Trp) ratio in vaginal fluids at different dpi. Pooled results from 3 different experiments. *P<0.05, IDO1-deficient *vs.* C57BL/6 mice at the days indicated. N.S., not significant. (**E**) Vaginal fungal growth (Log_10_ CFU/100 µl VF ± s.e.m.) at different dpi in mice (*n* = 6) treated intraperitoneally with a mixture of l-kynurenine, 3-hydroxykynurenine and 3-hydroxyanthranilic acid or PBS (None). Pooled data from 3 different experiments.*P<0.05, treated *vs.* untreated mice at the days indicated. N.S., not significant. (**F**) Periodic acid-Schiff-stained vaginal sections and inflammatory cell recruitment in vaginal fluids (May–Grünwald Giemsa staining in the insets) acquired with a 40× and 100× objective, respectively, at 21 dpi. Scale bars, 100 µm. Representative image from 3 experiments. (**G**) Cytokine levels (pg/mg, cytokine/total proteins for each sample, at 21 dpi) in the vaginal fluids of mice treated as above. Pooled data from 3 different experiments. *P<0.05, treated *vs.* untreated (None) mice. (**H**) Vaginal immunohistochemistry of naïve or infected mice at 3 days after re-challenge. Double staining was done with anti-IL-10-FITC and polyclonal rabbit to FoxP3 followed by anti-rabbit TRITC. Cell nuclei were stained with DAPI (blue). Representative pictures (out of 2 experiments) were taken with a 20× objective. Scale bars, 50 µm.

IDO1 is known to contribute to Treg cell function in mucosal candidiasis [13] and Treg cells are essential components of immune memory to the fungus [42]. We looked, therefore, for IDO1 protein and gene expressions, kynurenine-to-tryptophan ratios, a valid indicator of IDO1 activity [43], and IL-10-producing T cells in mice with VVC. We found that IDO1 was promptly induced in infection at the protein (Figure 4A) and gene (Figure 4B) expression levels, maintained elevated thereafter and was associated with increased levels of kynurenines, downstream products of IDO1 with immunoregulatory functions [44], [45] (Figure 4C) and increased kynurenine to tryptophan ratio [43] (Figure 4D). Both the kynurenine levels and the kynurenine-to-tryptophan ratio were lower in IDO1-deficient mice (Figure 4C and D). The kynurenines were functionally active as replacement therapy with a mixture of l-kynurenine, 3-hydroxykynurenine and 3-hydroxyanthranilic acid, all molecules downstream of the IDO1 pathway [35], restored immunoprotection to VVC, as indicated by restriction of fungal growth (Figure 4E), amelioration of tissue inflammation (Figure 4F), decreased IL-17A and increased IL-10 production at 21 dpi (Figure 4G). These data suggested that IDO1 mediates the production of tolerogenic kynurenines in VVC. To define the effector mechanism of tolerance in VVC, we evaluated IL-10 and FoxP3 expression in the vaginal parenchyma of re-infected mice. Immunostaining revealed the presence of cells expressing both IL-10 and FoxP3 in wild-type but not IDO1-deficient (Figure 4H), a finding consistent with the levels of IL-10 production (Figure 4G). Interestingly, the kynurenine levels were also lower in IDO1-deficient than wild-type mice after re-challenge (2.2±0.7 *vs.* 0.5±0.3, wild-type *vs.* IDO1-deficient mice, 3 days after re-challenge). Thus, IDO1, promptly induced in infection, is apparently required for local immunoregulation via IL-10^+^ Treg cells.

### Treg cells control the Th1/Th17 cell balance in murine VVC

**Figure 5:**
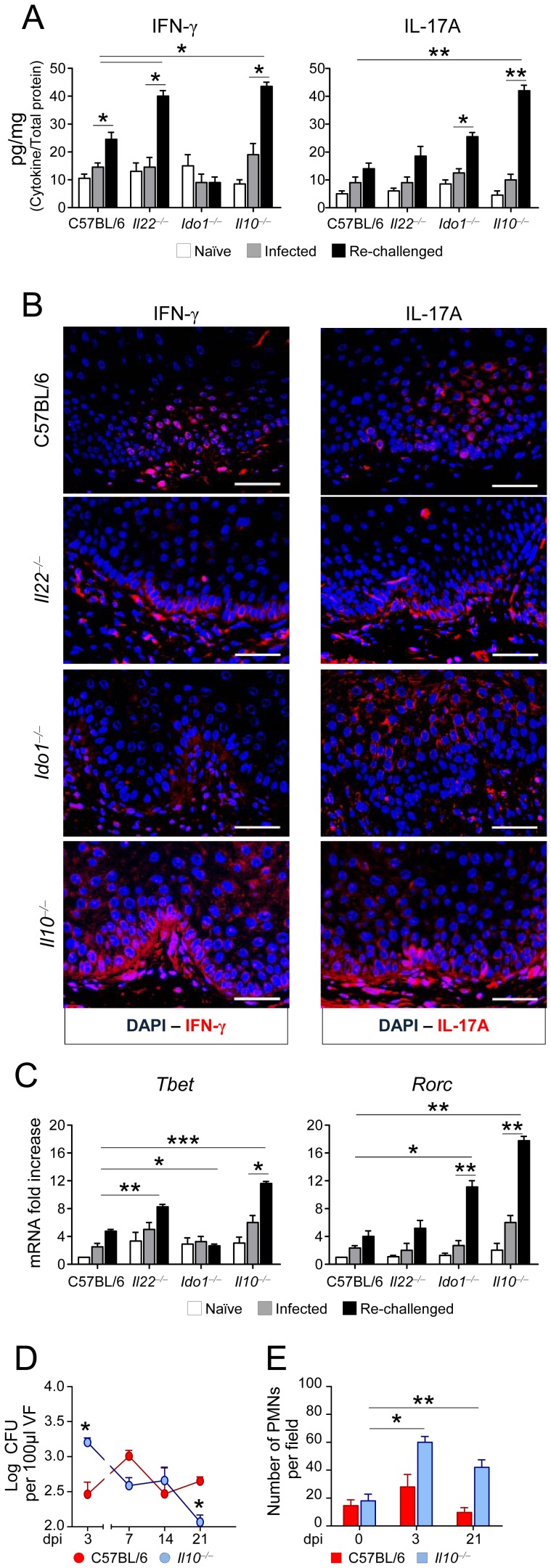
Treg cells control the Th1/Th17 cell balance in murine VVC. (**A**) IFN-γ and IL-17A production (pg/mg, cytokine/total proteins for each sample) in the vagina fluids of mice (*n* = 6) intravaginally inoculated with 5×10^6^ *C. albicans* blastoconidia and re-infected 3 weeks later. Assays were done at 3 days after the primary infection (infected) or re-challenge. Pooled data from 5 experiments. (**B**) Vaginal immunohistochemistry of re-infected mice. Staining was done with anti-IFN-γ-PE and anti-IL-17A-PE antibody. Cell nuclei were stained with DAPI (blue). Representative pictures (out of 2 experiments) were taken with a 20× objective. Scale bars, 200 µm. (**C**) Expression (RT-PCR) of transcript factor genes in purified CD4^+^ T cells from the draining lymph nodes. Assays were done at 3 days after the primary infection (infected) or re-challenge. *P<0.05, **P<0.01 and ***P<0.001, knockout *vs.* C57BL/6 mice at 3 days after re-challenge and re-challenged *vs.* infected mice. Control immunostaining performed on the vaginas from naïve mice revealed an increased expression of IL-17A in IL-17RA- and IL-10-deficient mice and of IFN-γ in IL-10-deficient mice. (**D**) Fungal growth in C57BL/6 or IL-10-deficient mice (*n* = 6) inoculated with 5×10^6^ *C. albicans* blastospores. Fungal growth was quantified in the vaginal fluids at different days post-infection (dpi) and expressed as Log_10_ CFU/100 µl VF ± s.e.m. (**E**) Polymorphonuclear cells (PMNs) quantification in the vaginal fluids at different dpi. PMNs were identified by nuclear morphology and enumerated per field at ×100 magnification. Pooled data from 3 experiments. *P<0.05 and **P<0.01, 3 or 21 *vs.* 0 dpi.

The increased resistance seen later in infection in IL-22-deficient but not IDO1-deficient mice, prompted us to define mechanisms of resistance that are independent from IL-22 but dependent on IDO1. We evaluated the production and expression of IFN-γ and IL-17A in the vagina and the expression of Th-specific transcription factor genes in purified CD4^+^ T cells from the draining lymph nodes of re-infected mice. We found that resistance to re-challenge in C57BL/6 or IL-22-deficient mice correlated with high-level production (Figure 5A) and expression (Figure 5B) of IFN-γ and IL-17A. IFN-γ but not IL-17A, production (Figure 5A) and expression (Figure 5B) were instead reduced in IDO1-deficient mice in which *Tbet* expression was also lower and *Rorc* expression higher as compared to wild-type mice (Figure 5C). Importantly, the production of IFN-γ and IL-17A by CD4^+^ T cells isolated from mice during the primary infection (3 dpi) was significantly lower than that observed upon re-challenge (Figure 5A). Therefore, these data indicated that an appropriate Th1/Th17 cell balance is required for the expression of acquired antifungal immunity. Studies in IL-10-deficient mice confirmed that IL-10^+^ Treg cells essentially control this balance. IL-10-deficient mice, although capable of restraining the fungal growth during the primary infection (Figure 5D) and after re-challenge (Figure S1F in Text S1), were unable to control tissue inflammation (Figure S1G in Text S1) and PMN recruitment (Figure 5E) during the infection, and this was associated with high-level production (Figure 5A) and expression (Figure 5B) of IFN-γ and IL-17A, and with high *Tbet* and *Rorc* expressions in CD4^+^ T cells (Figure 5C). Thus IL-10 is required to restrain immunopathology, to which Th17, more than Th1, cells contribute as revealed by subsequent studies in IFN-γ- or IL-17RA-deficient mice. While more resistant to the infection in the early stage– likely due to the high levels of IL-22 (Figure S2A in Text S1)–both IFN-γ- and IL-17RA-deficient mice progressively become susceptible to infection, as indicated by the failure to restrain fungal growth during the primary infection (Figure S2B in Text S1) or after re-challenge (Figure S2C in Text S1) and to limit inflammation (Figure S2D-F in Text S1). In contrast to IL-17A, the levels of IFN-γ were either absent or greatly reduced (Figure S2G in Text S1), a finding indicating that IFN-γ is a key mediator of acquired resistance to the fungus, to which IL-17RA signaling contributes, as already suggested [13]. Studies in IL-12p40-deficient mice confirmed the combined requirement of Th1 and Th17 cells for optimal antifungal memory resistance, as compared to IL-12p25-deficient or IL-23p19-deficient mice (data not shown).

### Immunity and tolerance in murine VVC occur through distinct innate recognition receptors

**Figure 6:**
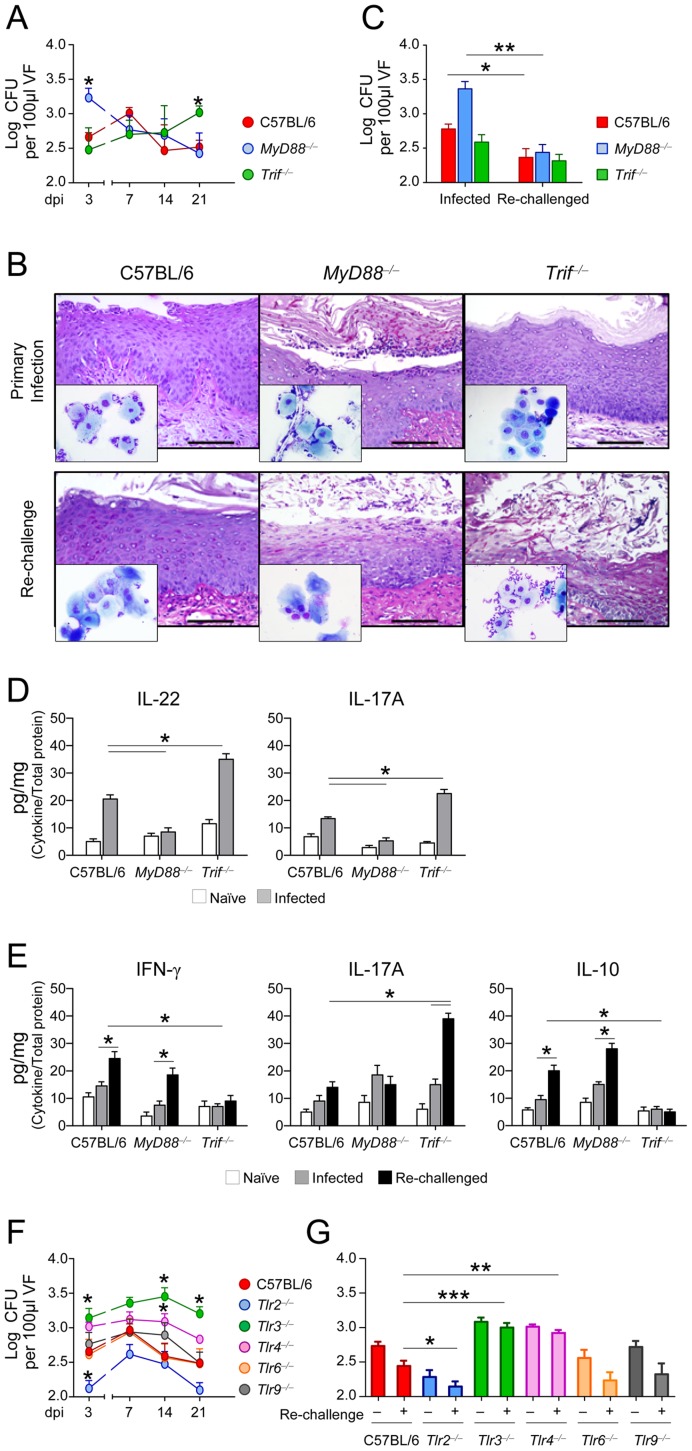
Immunity and tolerance to VVC occurs through distinct innate recognition receptors. (**A**) In vivo kinetics of fungal growth in the vagina of C57BL/6, MyD88- or TRIF- deficient mice (*n* = 6), inoculated intravaginally with 5×10^6^ *C. albicans* blastospores. Fungal growth was quantified at different dpi and expressed as Log_10_ CFU/100 µl VF ± s.e.m. Pooled data from 4 experiments. *P<0.05, MyD88- or TRIF-deficient *vs.* wild-type mice. (**B**) Periodic acid-Schiff-stained vaginal sections and inflammatory cell recruitment in vaginal fluids (May–Grünwald Giemsa staining in the insets) at 3 days after the primary infection or after re-challenge. Representative images of histology sections and vaginal fluids (out of 4 experiments) were acquired with a 40× and 100× objective respectively. Scale bars, 100 µm. (**C**) Fungal growth at 3 dpi in mice (*n* = 6) after the primary infection or re-challenge. Pooled data from 4 experiments. *P<0.05 and **P<0.01, Re-challenged *vs.* not re-challenged (infected) mice. (**D**) Cytokine levels (pg/mg, cytokine/total proteins for each sample, at 3 dpi) in the vaginal fluids. Pooled data from 4 experiments.*P<0.05 Knockout *vs.* C57BL/6 mice. (**E**) IFN-γ, IL-17A and IL-10 production (pg/mg, cytokine/total proteins) in the vaginal fluids of mice (*n* = 6) at 3 days after the primary infection (infected) or re-challenge. Pooled data from 4 experiments.*P<0.05, Knockout *vs.* C57BL/6 mice at 3 days after re-challenge and re-challenged *vs.* infected mice. (**F**) Fungal growth in C57BL/6 and TLR-deficient mice (*n* = 6) at different dpi. Pooled data from 3 experiments. *P<0.05, TLR-deficient *vs.* wild-type mice. (**G**) Fungal growth (at 3 dpi) in mice (*n* = 6) re-challenged 3 weeks after the primary infection. Pooled data from 3 experiments. *P<0.05, WT *vs.* KO mice.

We know that functional yet balanced reactivity to *C. albicans* at mucosal surfaces requires both the myeloid differentiation primary response gene 88 (MyD88) and the TIR-domain-containing adapter-inducing interferon-β (TRIF) as well as different upstream innate receptors [13]. We evaluated resistance to the primary infection and re-challenge in MyD88-or TRIF-deficient mice intravaginally infected with the fungus. Early in infection, fungal burden was higher in MyD88-deficient mice and declined thereafter (Figure 6A). The inflammatory response with PMN recruitment observed early in infection resolved later in infection (Figure 6B), at a time when those mice developed resistance to re-infection (Figure 6C). Growth was instead restrained in TRIF-deficient mice early in infection, but fungal growth was observed in the vagina at a later stage (Figure 6A), when mice effectively controlled fungal growth if re-challenged (Figure 6C), but not the associated inflammatory response (Figure 6B). Consistent with these findings, IL-22 and IL-17A were particularly lower in MyD88-deficient mice as compared to TRIF-deficient mice (Figure 6D), whereas IL-17A more than IFN-γ/IL-10 were present in TRIF-deficient mice (Figure 6E). To identify the upstream innate receptors involved in the response, we infected mice deficient in receptors known to recruit the MyD88 (TLR2/TLR6/TLR9), TRIF (TLR3), or both (TLR4) pathways and whose expression was observed in the vagina (Figure S3 in Text S1). Compared to wild-type mice, resistance to primary infection (Figure 6F) and to re-challenge (Figure 6G) was similar in TLR9-deficient or TLR6-deficient mice; it was greatly increased in TLR2-deficient mice but impaired in TLR3- or TLR4-deficient mice. Thus, the MyD88 pathway mainly contributes to antifungal mucosal resistance, through the involvement of TLR4, TLR3 (this study) and the beta-glucan receptor Dectin-1 [46], while the TRIF pathway contributes to tolerance via TLR3 and TLR4.

### Functional genetic variants in *IL22* and *IDO1* genes influence susceptibility to RVVC

**Figure 7:**
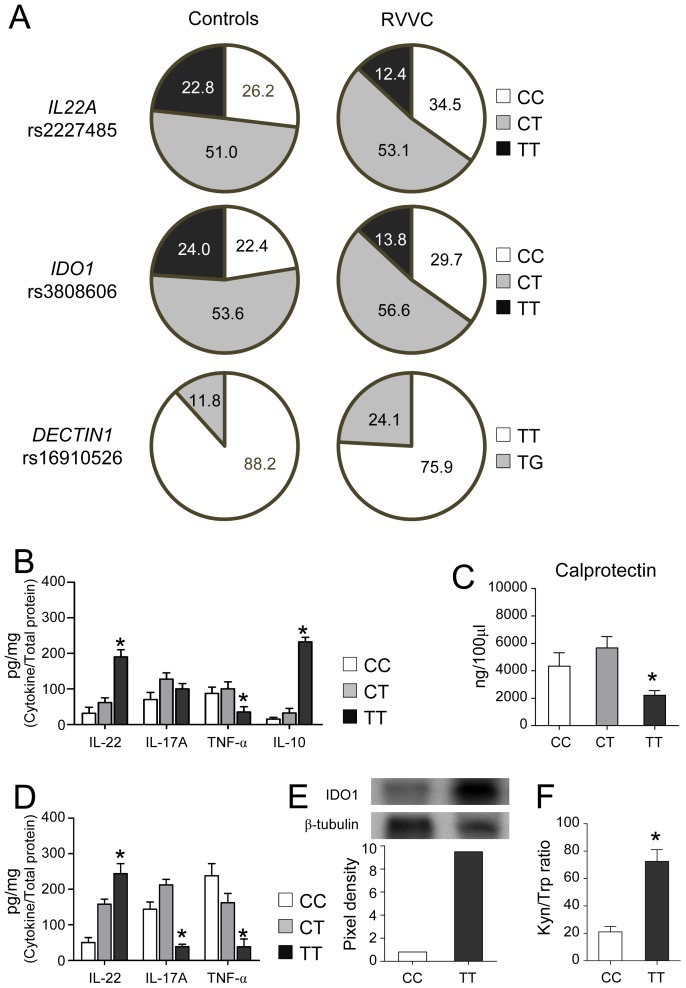
Functional genetic variants in *IL22* and *IDO1* genes influence susceptibility to RVVC. (**A**) Frequencies (%) of the different genotypes for single nucleotide polymorphisms (SNPs) in *IL22* (rs2227485), *IDO1* (rs3808606) and *DECTIN1* (rs16910526, Y238X) genes among controls (*n* = 263) and patients with RVVC (*n* = 145). (**B**) Cytokines (pg/mg, cytokine/total proteins) and (**C**) calprotectin levels (mean values ± s.e.m.) in the vaginal fluids of women bearing the CC, CT or TT genotypes at rs2227485 in *IL22*. (**D**) Cytokine levels (pg/mg, cytokine/total proteins, mean values ± s.e.m.) in the vaginal fluids of women bearing the CC, CT or TT genotypes at rs3808606 in *IDO1*. Data in B, C and D indicate mean values from measurements using samples obtained from at least 10 different women for each genotype, assessed in quadruplicates. (**E**) IDO protein expression in cells from the vaginal fluids of women bearing the CC or TT genotypes at rs3808606 in *IDO1*. Western blots of a representative sample out of 10 with similar results are shown along with the corresponding pixel density ratio normalized against β-tubulin. (**F**) Kynurenine-to-tryptophan (Kyn/Trp) ratios in vaginal fluids of women bearing the CC or TT genotypes at rs3808606 in *IDO1*. Pooled data from vaginal fluids obtained from at least 10 different women for each genotype. *P<0.05, TT *vs.* CC genotypes.

Because of the above results, we investigated whether genetic variants possibly affecting the functions of IL-22 and IDO1 might influence susceptibility to human VVC and RVVC (Table S1 in Text S1). Within the set of single nucleotide polymorphisms (SNPs) tested (Table S2 in Text S1), we found that the genotype frequencies for rs2227485 in *IL22* and rs3808606 in *IDO1* were significantly different between controls and RVVC, but not VVC, patients (Figure 7A and Table S3 in Text S1). Specifically, the TT genotype at rs2227485 in *IL22* was significantly associated with a decreased risk for RVVC [12.4% in RVVC *vs.* 22.8% in controls; odds ratio (OR), 0.48; 95% confidence interval (CI), 0.27–0.85; *P* = 0.01]. Likewise, the TT genotype at rs3808606 in *IDO1* also correlated with a minor susceptibility to RVVC (13.8% in RVVC *vs.* 24.0% of controls; OR, 0.51; 95% CI, 0.29–0.88; *P* = 0.02).

As dectin-1 mediates IL-22 production in mucosal candidiasis [46], and the early stop-codon mutation Y238X in *DECTIN1* (rs16910526) has been shown to predispose to familial RVVC in homozygous individuals [47], we also assessed the distribution of this substitution within our study subject group. As expected, we found that the TG genotype conferred an increased risk for RVVC (24.1% in RVVC *vs.* 11.8% in controls; OR, 2.38; 95% CI, 1.40–4.06, *P* = 0.001) (Figure 7A and Table S3 in Text S1). Functional analyses confirmed that protection afforded by carriage of the TT genotype at rs2227485 in *IL22* correlated with high levels of vaginal IL-22 and decreased levels of pro-inflammatory IL-17A, TNF-α and calprotectin (Figure 7B and C). Likewise, high levels of IL-22 and decreased levels of IL-17A and TNF-α were observed in women carrying the protective TT genotype at rs3808606 in *IDO1* (Figure 7D), and they were associated with enhanced IDO1 expression in vaginal cells (Figure 7E) and increased kynurenine-to-tryptophan ratio (Figure 7F). Altogether, these data provide evidence that common genetic variants leading to enhanced expression phenotypes of *IL22* and *IDO1* may contribute to protection in RVVC.

## Discussion

This study disentangles resistance and tolerance components of murine and human *C. albicans* vaginal infection and introduces the challenging notion of a disease due to a defective tolerance mechanism. While some degree of inflammation is required for protection, particularly at mucosal tissues during the transitional response occurring between the rapid innate and slower adaptive responses, progressive inflammation worsens disease and ultimately prevents pathogen eradication [24], [35]. Much like in gastrointestinal candidiasis [13], resistance and tolerance mechanisms in VVC are activated through the contribution of innate and adaptive immune responses, involving distinct modules of immunity, i.e., IL-22 and Th1/Th17 cells for resistance and IL-10^+^ Treg cells for tolerance. It has already been shown that IL-22 provides antifungal resistance through disparate mechanisms, including i) growth control of the early infecting morphotype, the yeasts, via the induction of antimicrobial peptides with anticandidal activity and ii)epithelial integrity control, via phosphorylation of STAT3 [13], known to be required for limiting damage and inflammation in mucosal candidiasis [48]. Thus IL-22 variations may explain why some patients are at high risk of vaginal yeast infection.

Although many different cell types produce IL-22 [41], intestinal ILCs expressing AhR, now termed ILC3 [49], are known to produce, in addition to other cytokines, IL-22 [50]. ILCs reflect many functions of CD4^+^ T helper cells but expand and act shortly after stimulation. They play fundamental roles early in response to infection and injury, in the maintenance of homeostasis, and possibly in the regulation of adaptive immunity [51]. We found here that NKp46^+^ cells expanded in the vagina in infection and produced IL-22, more than IL-17A, likely via AhR. As expression of cytokines by ILCs is regulated by signals provided by epithelial cells in response to microbiota, our finding may provide a mechanistic explanation for the link between microbial dysbiosis and vaginal candidiasis. Indeed, the fact that IL-22 production is driven by commensals may explain how antibiotic therapy and iatrogenic immunosuppression are major predisposing factors in candidiasis and, more generally, how the bacterial-fungal population dynamics impact on vaginal homeostasis and inflammatory diseases.

Mutations in *IL17F*, *IL17R* and *DECTIN1* in patients with chronic mucocutaneous candidiasis, as well as neutralizing autoantibodies against IL-17 and IL-22 in patients with autoimmune polyendocrinopathy-candidiasis-ectodermal dystrophy, directly impair IL-17 and IL-22 immunity [47], [52]–[54]. We found here that IL-22, more than IL-17A, contributes to resistance to VVC. In mice, resistance was abrogated under conditions of IL-22 or IL-17F, more than IL-17A, deficiency, whereas a common genetic variant in human *IL22* leading to enhanced production of IL-22, but not IL-17A and TNF-α, conferred protection against RVVC. Thus, in addition to functional polymorphisms in genes coding for mannose-binding lectin [55]–[57], IL-4 [58], and the inflammasome component NALP3 [59] in predisposing to RVVC, our study identified an *IL22* variant that is associated with a decreased risk for RVVC and is consistent with the findings obtained relative to *DECTIN1* deficiency [47].

To our knowledge, this is the first genetic variant in *IL22* found to be associated with a human disease [41], and our finding confirms the important functions of the IL-22–IL-22R pathway in regulating immunity, inflammation and tissue homeostasis, and the therapeutic potential of targeting this pathway in human disease [41].

Considered a master regulator of antifungal resistance and tolerance at mucosal surfaces [34], IDO1 has gained reputation in the field of immune mycology owing to its ability to generate immunomodulatory kynurenines that induce Treg cells suppressing local antifungal T-cell responses, thus favoring fungal persistence [34]. It is known that IDO1 activity exhibits relatively large interindividual variability, in particular under pathological conditions [60], [61] and that there are naturally occurring polymorphisms that transcriptionally regulate the human *IDO1* gene [62], [63]. Thus, genetic factors are involved in interindividual variability of IDO expression and/or activity. We found that an *IDO1* variant leading to enhanced IDO1 expression and concomitant production of kynurenines was associated with decreased risk for RVVC. IDO1 regulates tolerance to the fungus at the vaginal level, as it ameliorates immunopathology that positively correlated with the magnitude of the immune response. It was indeed required for the generation of IL-10^+^ Treg cells negatively affecting Th1/Th17 cells. Moreover, IDO1 also favored the induction of optimal antifungal memory resistance, an activity likely due to its ability to limit tissue damage, thus allowing for a higher magnitude and duration of the immune response than would have been otherwise possible. Finally, the finding that IL-22 was up-regulated in condition of IDO1 deficiency indicates that the resistance and tolerance mechanisms are to some extent reciprocally regulated.

Understanding the mechanisms that are critical for host survival is important for the choice of therapeutic approaches. Antifungal therapy is highly effective for individual symptomatic attacks but does not prevent recurrences. In addition to the associated costs that are very high [64], there is concern that repeated treatments might induce drug resistance. Thus medical treatments that increase host resistance, such as antibiotics, place selective pressures on pathogens. As tolerance mechanisms are not expected to have the same selective pressure on pathogens, new drugs that target tolerance will provide therapies to which low-virulence fungi, such as *C. albicans*, will not develop resistance. Kynurenines appear to fulfill this requirement.

## Material and Methods

### Ethics statement

Murine experiments were performed according to the Italian Approved Animal Welfare Assurance A-3143-01 and Legislative decree 245/2011-B regarding the animal license obtained by the Italian Ministry of Health lasting for three years (2011–2014). Infections were performed under avertin anesthesia and all efforts were made to minimize suffering. The experimental protocol was designed in conformity with the recommendations of the European Economic Community (86/609/CEE) for the care and the use of laboratory animals, was in agreement with the Good Laboratory Practices and was approved by the animal care Committee of the University of Perugia (Perugia, Italy). All patients and control subjects were observed at the Microbiology Department, S. Maria della Misericordia Medical Center (Perugia, Italy) and answered a detailed questionnaire reporting social and demographic information, medical history, subjective symptoms for gynecological infections and sexual behavior. The study approval was provided by the University ethics committee (Prot. 2012-028) and informed written consent was obtained from all participants. Enrollment took place between January 2009 and June 2012.

### Mice

Female C57BL/6 and NOD.SCID (NOD.CB17-*Prkdc* ^scid^/NCrCrl) mice, 8–10 wk old, were purchased from Charles River (Calco, Italy). Homozygous *Il22^−/−^, Ido1^−/−^, Ahr^−/−^, Ifng^−/−^, Il17ra^−/−^, Il17a^−/−^, Il17f^−/−^, Il10^−/−^, MyD88^−/−^, Trif^−/−^, Tlr2^−/−^, Tlr3^−/−^, Tlr4^−/^, Tlr6^−/−^ and Tlr9^−/−^* mice on the C57BL/6 background were bred under specific pathogen-free conditions at the Animal Facility of the University of Perugia, Perugia, Italy.

### Fungal strains, vaginal infection and treatments

Mice were treated subcutaneously with 0.1 mg of β-estradiol 17–valerate (Sigma Chemical Co.) dissolved in 100 µl of sesame oil (Sigma) 48 h before vaginal infection. Estrogen administration continued weekly until completion of the study to maintain pseudoestrus. The estrogen-treated mice were inoculated intravaginally with 20 µl of phosphate-buffered saline (PBS) suspension of 5×10^6^ viable *C. albicans* 3153A blastospores from early–stationary-phase cultures (i.e., 18 h of culture at 36°C in Sabouraud-dextrose agar with chloramphenicol plates, BD Diagnostics). Re-challenge was performed by intravaginal inoculation of 5×10^6^ blastospores, 3 or 5 weeks after the primary infection. In the vaccine-induced resistance experiments, 5×10^6^ heat-killed *C. albicans* (HCA), obtained by exposing 1×10^8^ cells/ml at 56°C for 30 min, or live low-virulence mutant cells obtained by mutagenesis [65] were intravaginally injected into estradiol-treated mice, 3 weeks before re-challenge. Control mice received PBS. The time course of infection was monitored in individual mice by culturing 100 µl of serially diluted (1∶10) vaginal lavages on Sabouraud-dextrose agar with chloramphenicol plates. Vaginal lavages were conducted using 100 µl of sterile PBS with repeated aspiration and agitation. CFUs were enumerated after incubation at 36°C for 24 h and expressed as log_10_ CFU/100 µl of lavage fluid. Quantitative counts of CFU in lavage fluids were evaluated successively in mice anesthetized for each lavage. Cytospin preparations of the lavage fluids were stained with May-Grünwald-Giemsa and observed with a BX51 microscope equipped with a high-resolution DP71 camera (Olympus). IL-22 blockade was achieved as described [13] by intraperitoneally injecting mice with a total of 300 µg of mAb neutralizing IL-22 (clone AM22.3, mouse IgG2a) or isotype control mAb (Sigma-Aldrich) the day of and 1 day after the infection. Recombinant murine IL-22 (BioLegend) was given intravaginally (1 µg/mouse) daily, the day of the infection and 1 and 2 days after. Control mice received PBS. Mice were treated intraperitoneally daily, starting 3 days before and up to 3 days after the infection with PBS or 20 µg/kg of a mixture of l-kynurenine, 3-hydorxykynurenine, and 3-hydroxyanthranilic acid (Sigma-Aldrich).

### Cell isolation

For isolation of CD4^+^ cells, iliac and inguinal lymph nodes were aseptically removed and cut into small pieces in cold medium. The dissected tissue was then incubated in medium containing collagenase XI (0.7 mg/ml; Sigma-Aldrich) and type IV bovine pancreatic DNase (30 mg/ml; Sigma-Aldrich) for 30–45 min at 37°C. A single cell suspension was obtained and incubated with CD4 MicroBeads (Miltenyi Biotech) before magnetic cell sorting. Vaginal tissues were chopped into fragments and incubated in 1.3 mM EDTA for 30 min at 37°C, as described [66], followed by digestion for 90 min in collagenase type XI (1 mg/ml). These digested pieces were minced and filtered through a nylon mesh, and the resulting cells were filtered through a 70-µm filter. NKp46^+^ and γδ T cells were purified as per the manufacturer's instruction, with the mouse anti-NKp46 MicroBead Kit and the TCR γδ T cell isolation kit (Miltenyi Biotec).

### Flow cytometry and intracellular staining

Antibodies were as follows: anti-CD3ε (145-2C11), anti-γδ (GL3) (BD PharMingen) and anti-NKp46 (CD335) (eBioscience). All staining reactions were performed at 4°C on cells first incubated with an Fc receptor mAb (2.4G2) to reduce non-specific binding. For intracellular staining, cells were stimulated with PMA/ionomycin, added of brefeldin and then permeabilized with the CytoFix/CytoPerm kit (BD Biosciences) for intra-cytoplasmic detection of IL-17A (clone: eBio17B7, eBioscience), and IL-22 (clone 14.03.01, R&D System). Cells were analyzed with a FACScan flow cytofluorometer (Becton Dickinson) equipped with CELLQuest software.

### Histological analysis

For histology, the vaginas were removed and immediately fixed in 10% neutral buffered formalin (Bio-optica, Milan) for 24 h. The vaginas were dehydrated, embedded in paraffin, sectioned into 3–4 µm and stained with periodic acid-Schiff reagent. Histology sections were observed using a BX51 microscope equipped with a high-resolution DP71 camera (Olympus).

### ELISA and real-time PCR

The level of murine and human cytokines in the lavage fluids were determined by Kit ELISA (R&D Systems). The detection limits of the assays were <10 pg/ml for IL-22, <3 pg/ml for IL-10, <30 pg/ml for IL-17F, <10 pg/ml for IL-17A and <1.6 pg/ml for IFN-γ for murine cytokines and <2.7 pg/ml for IL-22, <15 pg/ml for IL-17A, <15 pg/ml for TNF-α and <4 pg/ml for IL-10 for human cytokines. Data were normalized to total protein levels for each sample as determined using the Bio-Rad Protein assay (Life Science, Bio-Rad Laboratories S.r.l. Milan, Italy) and expressed as pg cytokine/mg total protein. Results represent mean cytokine levels (± s.e.m.) from samples pooled from two similar experiments (*n* = 3–4 total samples per group). Human and murine calprotectin were determined by ELISA (Hycult biotech, Uden, The Netherlands and Immundiagnostik AG, Bensheim, Germany, for human and murine detection, respectively). Real-time PCR was performed using the iCycler iQ detection system (Bio-Rad) and SYBR Green chemistry (Finnzymes Oy). Vaginas and purified cells from lymph nodes were lysed and total RNA was extracted using RNeasy Mini Kit (QIAGEN, Milan, Italy) and was reverse transcribed with Sensiscript Reverse Transcriptase (QIAGEN) according to the manufacturer's directions. The PCR primers were as described [13]. Amplification efficiencies were validated and normalized against *Gapdh*. The thermal profile for SYBR Green real-time PCR was at 95°C for 3 min, followed by 40 cycles of denaturation for 30 s at 95°C and an annealing/extension step of 30 sec at 60°C. Each data point was examined for integrity by analysis of the amplification plot. The mRNA-normalized data were expressed as relative mRNA in knockout *vs.* wild-type mice and infected *vs.* naïve mice. TLR expression was evaluated in vaginal lysates using TLR specific primers and conditions as described [67]. The normalized CT value for the target amplification (Δ*C*_T_, *Tlr*) was determined by subtracting the average *Gapdh C*_T_ value from the average *Tlr C*_T_ value.

### Immunohistochemistry

The vagina was removed and fixed in 10% phosphate-buffered formalin, embedded in paraffin and sectioned at 5 mm. Sections were then rehydrated and after antigen retrieval in Citrate Buffer (10 mM, pH 6), sections were blocked with 5% BSA in PBS and stained with PE anti-IL-17A,-IFN-γ (XMG1.2),-IL-22, FITC-anti-NKp46 (eBioscience) followed by anti-rabbit TRITC (Sigma). Double staining with FITC anti-IL-10 (JES5-16E3) and rabbit polyclonal to FOXP3 (abcam) was followed by anti-rabbit TRITC. All mAbs were incubated overnight at 4°C. Images were acquired using a fluorescence microscope (BX51 Olympus) with a 20× objective and the analySIS image processing software (Olympus). 4′-6-Diamino-2-phenylindole (DAPI, Molecular Probes) was used to counterstain tissues and to detect nuclei.

### Western blotting

Cells from murine vaginas or from human vaginal washes were lysed in 2× Laemmli buffer (Sigma-Aldrich) and the lysates were separated in 10% Tris/glycine SDS gel and transferred to a nitrocellulose membrane. Blots of cell lysates were incubated with mouse anti-human IDO1 antibody clone 10.1 (Millipore Billerica) or rabbit polyclonal anti-murine IDO1 antibodies [61]. Normalization was performed by probing the membrane with mouse-anti-β-tubulin antibody (Sigma-Aldrich). Images were acquired with LiteAblotPlus chemiluminescence substrate (Euroclone S.p.A.) using ChemiDoc XRS and Imaging system (Bio-Rad Laboratories) and quantification was obtained by densitometry image analysis using Image Lab 3.1.1 software (Bio-Rad).

### Tryptophan and kynurenine assay

The kynurenine to tryptophan ratio was calculated by relating concentrations of kynurenine and tryptophan determined by HPLC with the internal calibrator 3-nitro-L-tyrosine, as described [43].Chromatography was performed on reversed-phase cartridges LiChroCART RP_18_ columns, tryptophan was monitored via its fluorescence at 285 nm excitation and 365 nm emission wavelengths, kynurenine was measured by its UV absorption at 360 nm wavelength. The kynurenine to tryptophan ratio (Kyn/Trp) was calculated and expressed as µmol kynurenine/mmol tryptophan.

### Patients

The study population included Caucasian women who had ≥4 (n = 145) or 1–3 (n = 293) culture-verified symptomatic episodes of a vulvovaginal *Candida* infection during a 12-month period (Table S1 in Text S1). Control subjects consisted of 263 age-matched healthy Caucasian women with no gynecologic complaints, no history of vaginal *Candida* infection, and who were currently culture-negative for vaginal pathogens. Exclusion criteria were pregnancy, diabetes mellitus, endocrine or immune deficiency disorders, use of immunosuppressive medications, antibiotics or high estrogen content contraceptives, chemotherapy or prior hysterectomy.

### DNA isolation and SNP genotyping

Cervicovaginal samples were obtained from all participants by instilling 3 ml of sterile saline into the posterior vagina, mixing the saline with secretions and withdrawing the solution with a syringe. All vaginal washes were centrifuged at 12,000× g for 10 min to separate the mucus from the PBS wash solution shortly after collection and pellet fractions and immediately frozen at −20°C. Genomic DNA was isolated from the pellet fraction using the QIAamp DNA Mini kit (Qiagen). SNPs were selected either from the literature or based on their ability to tag surrounding variants in the HapMap-CEU population of the International HapMap project, NCBI build B36 assembly HapMap phase III (http://www.hapmap.org). The Haploview 4.2 software was used to select haplotype-based tagging SNPs by assessing linkage disequilibrium blocks from the genes of interest with a pairwise correlation coefficient *r^2^* of at least 0.80 and a minor allele frequency of ≥5% in the HapMap-CEU population. SNPs evaluated are indicated in Table S2 in Text S1. Genotyping was performed as previously described [68], [69]. Primer sequences are available upon request. Each genotyping set comprised randomly selected replicates of sequenced samples and negative controls. Concordant genotyping was obtained for >99% assays.

For the functional assays, and unless stated otherwise, measurements were performed in vaginal washes obtained from at least 10 different women without ongoing symptoms and that had negative culture results for each genotype under study, assessed in quadruplicates.

### Statistical analysis

Student's T-test or analysis of variance (ANOVA) with Bonferroni's adjustment were used to determine statistical significance (P<0.05). The data reported are either from one representative experiment out of three to five independent experiments (western blotting and RT–PCR) or pooled from three to five experiments, otherwise. The in vivo groups consisted of 6–8 mice/group. Data were analyzed by GraphPad Prism 4.03 program (GraphPad Software). Genotype distributions among controls and VVC and RVVC patients were analyzed by Fisher's exact test and P values less than 0.05 were considered as significant. Genotype frequencies were distributed according to the Hardy-Weinberg equilibrium for all SNPs (P>0.05).

## Supporting Information

Text S1 **Supporting information.** Figure S1. Memory protective immunity in vaginal candidiasis. Figure S2. Th1 and Th17 cells mediate resistance to re-challenge. Figure S3. TLR expression in the vagina of C57BL/6 naïve mice. Table S1. Demographics and vaginal symptoms of study subjects. Table S2. Single nucleotide polymorphisms (SNPs) in *IL22*, *IDO1* and *DECTIN1* genes. Table S3. SNPs in *IL22*, *IDO1* and *DECTIN1* genes associated with VVC or RVVC.
(DOC)
