# Supplementary material for: IL-22 and IDO1 Affect Immunity and Tolerance to Murine and Human Vaginal Candidiasis
Source: PLoS Pathog. 2013 Jul 11;9(7):e1003486. doi: 10.1371/journal.ppat.1003486 (PMC3708875; doi:10.1371/journal.ppat.1003486)
Supplement: Text S1 — Supporting information. Figure S1. Memory protective immunity in vaginal candidiasis. Figure S2. Th1 and Th17 cells mediate resistance to re-challenge. Figure S3. TLR expression in the vagina of C57BL/6 naïve mice. Table S1. Demographics and vaginal symptoms of study subjects. Table S2. Single nucleotide polymorphisms (SNPs) in IL22, IDO1 and DECTIN1 genes. Table S3. SNPs in IL22, IDO1 and DECTIN1 genes associated with VVC or RVVC. (DOC) [file ppat.1003486.s001.doc]

**IL-22 and IDO1 AFFECT immunity and tolerance TO murine AND HUMAN vaginal candidiasis**

Antonella De Luca^1,4^ Agostinho Carvalho,^1^ Cristina Cunha,^1^ Rossana G. Iannitti,^1^ Lucia Pitzurra,^1,2^ Gloria Giovannini,^1^ Antonella Mencacci,^1,2^ Lorenzo Bartolommei,^1^ Silvia Moretti,^1^ Cristina Massi-Benedetti,^1^ Dietmar Fuchs,^3^ Flavia de Bernardis,^4^ Paolo Puccetti^1^ and Luigina Romani^1*^

**Supporting Information**

**Text S1**

**Figure S1.** Memory protective immunity in vaginal candidiasis.

**Figure S2.** Th1 and Th17 cells mediate resistance to re-challenge.

**Figure S3.** TLR expression in the vagina of C57BL/6 naïve mice.

**Table S1.** Demographics and vaginal symptoms of study subjects.

**Table S2.** Single nucleotide polymorphisms (SNPs) in *IL22*, *IDO1* and *DECTIN1* genes.

**Table S3.** SNPs in *IL22*, *IDO1* and *DECTIN1* genes associated with VVC or RVVC.

**Supplemental Figures Legend**

**Figure S1. Memory protective immunity in vaginal candidiasis.** (**A**)Fungal growth (3 dpi) in mice (*n = 6*) re-challenged 21 days after the primary infection with live 5 x 10^6^ *C. albicans* blastspores. Pooled data from 5 experiments.**P*< 0.05, ***P*< 0.01 and ****P*< 0.001, re-challenged *vs.* not-rechallenged (i.e, only infected) mice. Fungal growth (**B**) and vaginal histology (**C**) (3 dpi) in mice (*n = 6*) pretreated with heat-inactivated *C. albicans* (HCA), or the low-virulence (Vir^-^) mutant and infected 21 days as in (A). Vir^+^, mice infected as in (**A**) and re-infected 35 days later. Shown in the insets is the inflammatory cell recruitment in vaginal fluids (May–Grünwald Giemsa staining). Ct, control mice, i.e., mice infected only (3dpi). Pooled data or representative (histology) from 5 experiments. Histology and vaginal images were acquired with a 40 × and 100 × objective respectively. **P*< 0.05, re-challenged mice *vs*. infected mice (Ct). Scale bars, 100 µm. Fungal growth (**D**) and vaginal histology (**E**) (3 dpi) in NOD.SCID mice and the respective wild type (*n = 6*) pretreated with HCA and infected 21 days as in (**A**). **P*< 0.05, re-challenged mice *vs*. only infected mice (Ct). Shown in the insets is the inflammatory cell recruitment in vaginal fluids. Ct, control mice, i.e., mice infected only (3dpi). Pooled data or representative (histology) from 2 experiments. Histology and vaginal images were acquired with a 40 × and 100 × objective respectively. Scale bars, 100 µm. (**F**) Fungal growth (3dpi) in mice *(n = 6*) infected only or re-challenged 21 days after the primary infection. Pooled data from 3 experiments. **P*< 0.05 and ****P*< 0.001, Re-challenged *vs.* infected mice. (**G**) Periodic acid-Schiff-stained vaginal sections and inflammatory cell recruitment in vaginal fluids (May–Grünwald Giemsa staining in the insets) in the primary infection (Ct) or after re-challenge (3 dpi). Representative images (out of 3 experiments) were acquired with a 40 × and 100 × objective respectively. Scale bars, 100µm.

**Figure S2. Th1 and Th17 cells mediate resistance to re-challenge.** (**A**) C57BL/6, IFN-γ- or IL-17RA-deficient mice (*n* = 6) were intravaginally inoculated with 5 x 10^6^ *C. albicans* blastoconidia and cytokine levels (pg/mg, cytokine/total proteins for each sample) in the vaginal fluids at 3 dpi. (**B**) In vivo kinetics of fungal growth (quantified in the vaginal fluids of infected mice at different days post-infection (dpi) and expressed as Log_10_ CFU/100µl ± s.e.m.). **P*< 0.05, knockout *vs.* wild-type mice at the days indicated. (**C**) Fungal growth (3 dpi) in mice re-challenged 21 days after the primary infection. **P*< 0.05, ***P*< 0.01 and ****P*< 0.001, re-challenged *vs.* not-rechallenged (-) mice. (**D**) Polymorphonuclear cells (PMNs) quantification in the vaginal fluids at different dpi. PMNs were identified by nuclear morphology and enumerated per field at × 100 magnification. Each point represents an individual mouse, and horizontal bar indicates the means. ***P*< 0.01 and ****P*< 0.001, knockout *vs.* wild-type mice at the indicated days. (**E**) *S100a8* and *S100a9* mRNA expression (RT-PCR) in vaginal tissue at different dpi. *S100a8* and *S100a9* mRNA expression was quantified and normalized to mRNA expression of naïve mice. **P*< 0.05 and ***P*< 0.01, knockout *vs.* wild-type mice at the indicated days. (**F**) Histological analysis of periodic acid-Schiff-stained vaginal sections and inflammatory cell recruitment in vaginal fluids (May–Grünwald Giemsa staining in the insets) 3 days after the primary infection or re-challenge. Representative images of histology sections and vaginal fluids were acquired with a 40 × and 100 × objective respectively. Scale bars, 100 µm. (**G**) Cytokine levels (pg/mg, cytokine/total proteins for each sample) in the vaginal fluids in the primary infection (3 dpi). **P*< 0.05 and ***P*< 0.01, Knockout *vs.* wild-type mice.

**Figure S3. TLR expression in the vagina.** TLR mRNA expression in the vagina from C57BL/6 naïve mice (*n* = 3). TLR expression was evaluated by RT-PCR The normalized CT value for the target amplification (Δ*C*_T_,*Tlrs*) was determined by subtracting the average *Gapdh* *C*_T_ value from the average *Tlrs* *C*_T_ value.

**Figure S1**


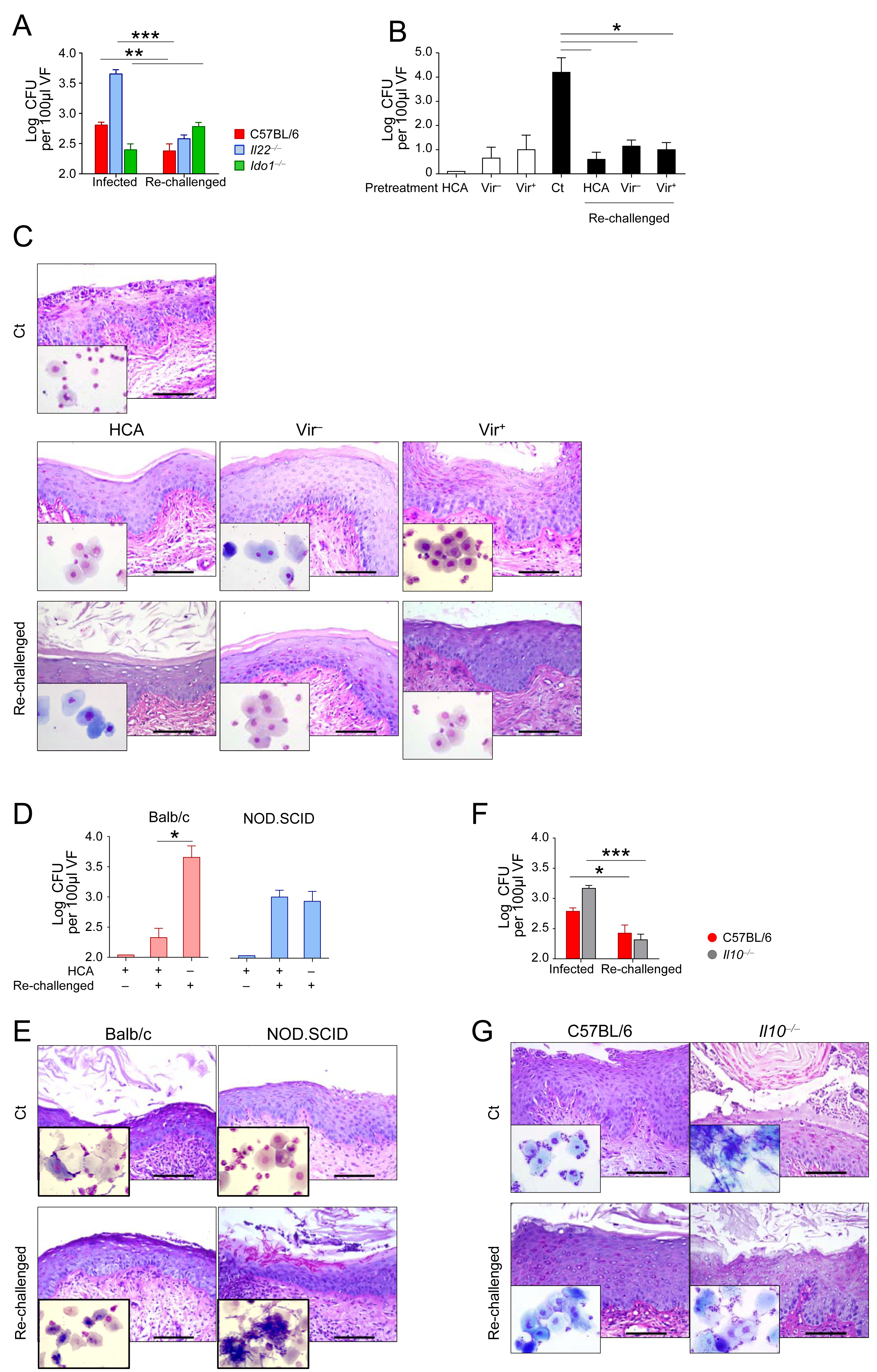


**Figure S2**


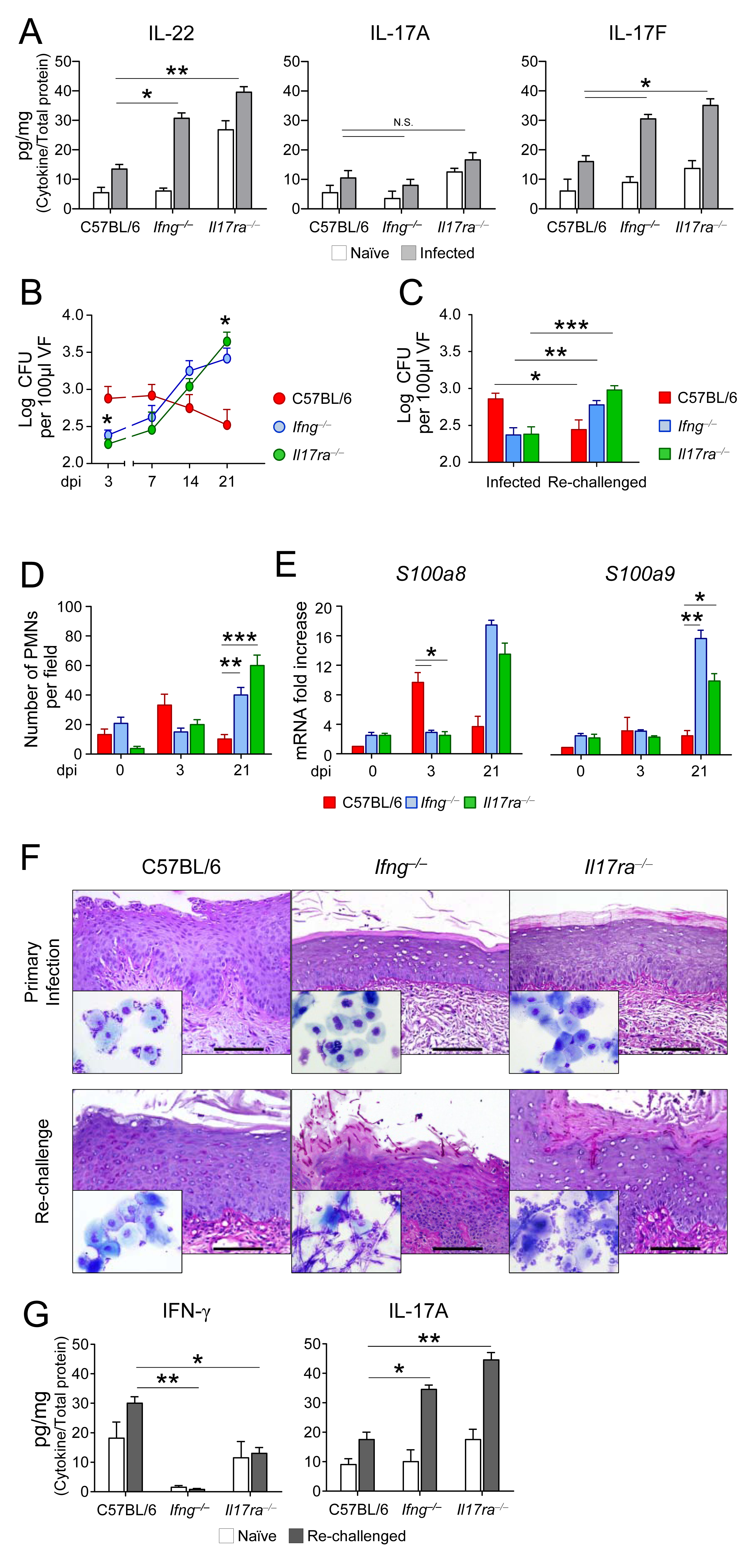


**Figure S3**

**
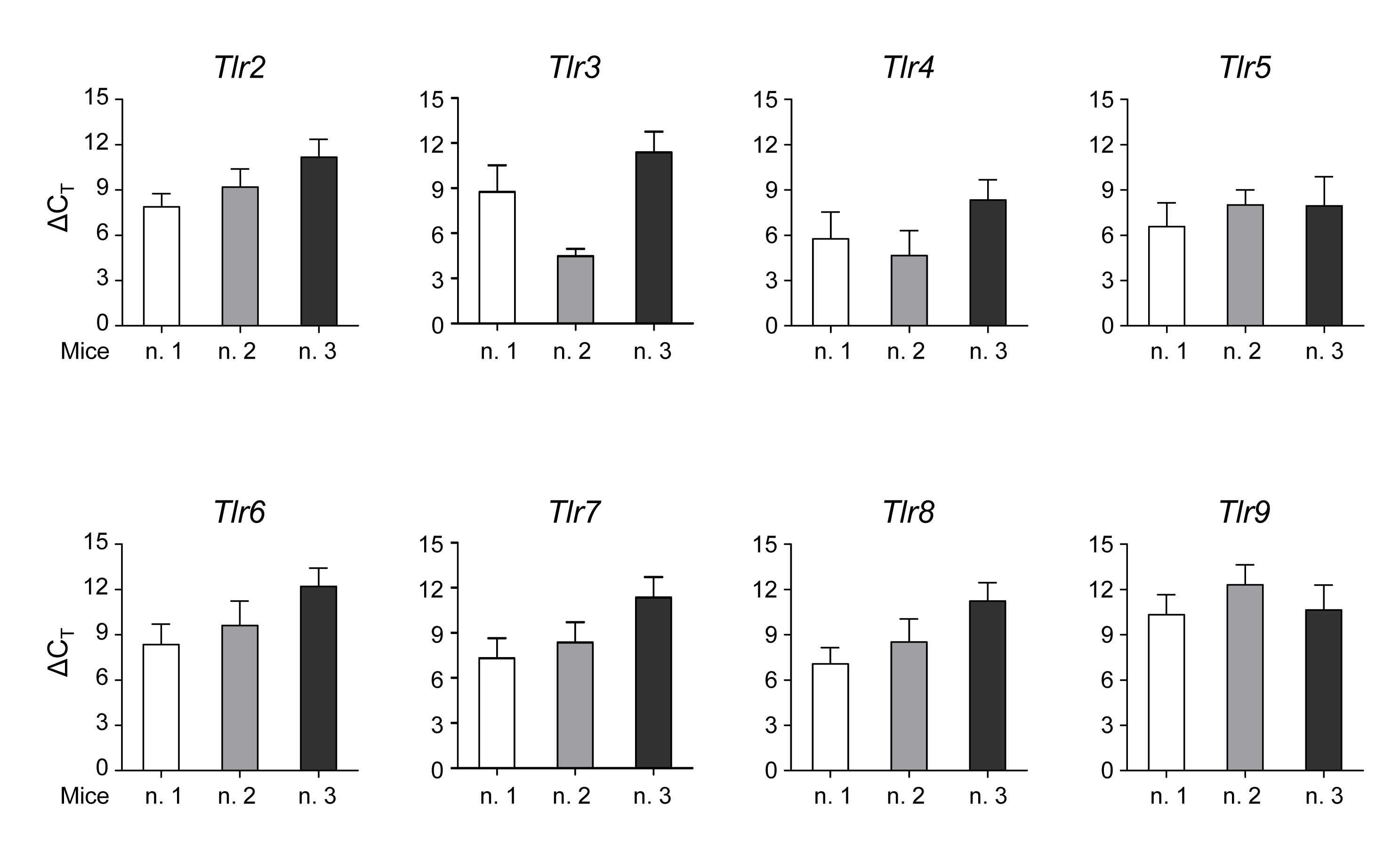
**

**Table S1.** Demographics and vaginal symptoms of study subjects.

| **Characteristic** | **Controls**  **(n=263)** | **VVC (n=293)** | **rVVC**  **(n=145)** |
| --- | --- | --- | --- |
| **Age, years** **[mean (range)]** | 31 (18-51) | 32 (19-50) | 31 (18-52) |
| ***Candida* infection** |  |  |  |
| *C. albicans* | 0 | 216 (73.7) | 106 (68.0) |
| *C. glabrata* | 0 | 40 (13.7) | 32 (20.5) |
| Others | 0 | 37 (12.6) | 18 (11.5) |
| **Number of episodes** | 0 | 1.7 ± 0.4 | 4.6 ± 0.8 |
| **Symptomatology** |  |  |  |
| Abnormal vaginal discharge | 0 | 184 (62.8) | 110 (70.5) |
| Vaginal or vulvar erythema | 0 | 72 (24.6) | 97 (62.2) |
| Vaginal fissures and/or excoriations | 0 | 67 (22.9) | 83 (53.2) |
| **Vaginal PMN counts** |  |  |  |
| Absent | 120 (23.3) | 31 (10.6) | 10 (6.4) |
| Present | 336 (65.1) | 179 (61.1) | 104 (66.7) |
| Numerous | 60 (11.6) | 83 (28.3) | 42 (26.9) |

VVC, vulvovaginal candidiasis; rVVC, recurrent VVC; PMN, polymorphonuclear neutrophils.

Unless otherwise stated, values are given as means ± standard deviation or n (%).

**Table S2.** Single nucleotide polymorphisms (SNPs) in *IL22*, *IDO1* and *DECTIN1* genes.

| **Gene** | **SNP rs number** | **Location** | **Base change** | **Aminoacid change** | **Minor allele** | **Additional SNPs tagged** |
| --- | --- | --- | --- | --- | --- | --- |
| ***IL22*** |  |  |  |  |  |  |
|  | rs2227485 | Near gene 5’ | C/T | None | C | rs2227491,rs2227483,rs1179246,rs10784699 |
|  | rs2046068 | Intron | A/C | None | C | rs2227478 |
|  | rs1179249 | Intron | A/C | None | A | rs1179250 |
|  | rs2227492 | Intron | A/G | None | A | None |
|  | rs1179251 | Intron | C/G | None | G | None |
|  | rs2227484 | Near gene 5’ | C/T | None | T | None |
|  | rs2227513 | Near gene 5’ | A/G | None | G | None |
| ***IDO1*** |  |  |  |  |  |  |
|  | rs7820268 | Intron | C/T | None | T | rs10089084,rs10108662,rs10089078 |
|  | rs3808606 | Near gene 5’ | C/T | None | T | rs3824259 |
|  | rs3739319 | Intron | A/G | None | A | rs11992749 |
| ***DECTIN1*** |  |  |  |  |  |  |
|  | rs16910526 | Exon | T/G | Y238X | G | None |

SNPs were selected either from the literature or based on their ability to tag surrounding variants in the HapMap-CEU population of the International HapMap project with a pairwise correlation coefficient *r^2^* of at least 0.80 and a minor allele frequency of ≥5%.

**Table S3.** SNPs in *IL22*, *IDO1* and *DECTIN1* genes associated with VVC or RVVC.

| **Gene**  **(SNP rs number)** | **Genetic model** | **Genotype** | **Controls (N=263)** | **VVC (N=293)** | | | **RVVC (N=145)** | | |
| --- | --- | --- | --- | --- | --- | --- | --- | --- | --- |
|  |  |  | **n (%)** | **n (%)** | **OR (95% CI)** | ***P*** | **n (%)** | **OR (95% CI)** | ***P*** |
| *IL22A*  (rs2227485) | Co-dominant | CC | 69 (26.2) | 87 (29.7) |  | 0.514 | 50 (34.5) |  | **0.023** |
|  |  | CT | 134 (51.0) | 150 (51.2) |  |  | 77 (53.1) |  |  |
|  |  | TT | 60 (22.8) | 56 (19.1) |  |  | 18 (12.4) |  |  |
|  |  |  |  |  |  |  |  |  |  |
|  | Dominant | CC | 69 (26.2) | 87 (29.7) | 0.84 (0.58-1.22) | 0.395 | 50 (34.5) | 0.68 (0.44-1.05) | 0.088 |
|  |  | CT+TT | 194 (73.8) | 206 (70.3) |  |  | 95 (65.5) |  |  |
|  |  |  |  |  |  |  |  |  |  |
|  | Recessive | CC+CT | 203 (77.2) | 237 (80.9) | 0.80 (0.53-1.20) | 0.297 | 127 (87.6) | 0.48 (0.27-0.85) | **0.012** |
|  |  | TT | 60 (22.8) | 56 (19.1) |  |  | 18 (12.4) |  |  |
|  |  |  |  |  |  |  |  |  |  |
| *IDO1*  (rs3808606) | Co-dominant | CC | 59 (22.4) | 87 (29.7) |  | 0.141 | 43 (29.7) |  | **0.033** |
|  |  | CT | 141 (53.6) | 146 (49.8) |  |  | 82 (56.6) |  |  |
|  |  | TT | 63 (24.0) | 60 (20.5) |  |  | 20 (13.8) |  |  |
|  |  |  |  |  |  |  |  |  |  |
|  | Dominant | CC | 59 (22.4) | 87 (29.7) | 0.68 (0.47-1.00) | 0.054 | 43 (29.7) | 0.69 (0.43-1.09) | 0.121 |
|  |  | CT+TT | 204 (77.6) | 206 (70.3) |  |  | 102 (70.3) |  |  |
|  |  |  |  |  |  |  |  |  |  |
|  | Recessive | CC+CT | 200 (76.0) | 233 (79.5) | 0.82 (0.55-1.22) | 0.357 | 125 (86.2) | 0.51 (0.29-0.88) | **0.020** |
|  |  | TT | 63 (24.0) | 60 (20.5) |  |  | 20 (13.8) |  |  |
|  |  |  |  |  |  |  |  |  |  |
| *DECTIN1*  (rs16910526) |  | TT | 232 (88.2) | 248 (84.6) | 1.36 (0.83-2.22) | 0.266 | 110 (75.9) | 2.38 (1.40-4.06) | **0.001** |
|  |  | TG | 31 (11.8) | 45 (15.4) |  |  | 35 (24.1) |  |  |

SNP – single nucleotide polymorphism; VVC – vulvovaginal candidiasis; RVVC – recurrent VVC; OR – odds ratio.

No genetic models were tested for the rs16910526 SNP in *DECTIN1*, as no individuals with the GG genotype were found.

Bold *P* values denote statistical significance.
